# Examining the relationship between big data analytics capabilities and organizational ambidexterity in the Malaysian banking sector

**DOI:** 10.3389/fdata.2023.1036174

**Published:** 2023-03-17

**Authors:** Norzalita Abd Aziz, Fei Long

**Affiliations:** ^1^UKM-Graduate School of Business, The National University of Malaysia (UKM), Bangi, Malaysia; ^2^Business School, Guangdong Ocean University, Yangjiang, Guangdong, China

**Keywords:** big data analytics capabilities, exploration, exploitation, ambidexterity, bank, Malaysia

## Abstract

Drawing on previous literature on dynamic capability view (DCV), we examine the effects of data analytics capabilities (BDAC) on organizational ambidexterity and the paradoxical tensions between exploration and exploitation in the Malaysian banking sector. Although banks are often considered as mature commercial organizations, they are not free of issues concerning technological advancement and organizational changes for long-term competitiveness. Through statistical analysis by using data from 162 bank managers in Malaysia, it is confirmed that BDAC positively influences the two contradictory aspects of organizational ambidexterity (i.e., explorative dynamic capabilities and exploitative dynamic capabilities), and explorative dynamic capabilities also mediate the positive relationship between BDAC and exploitative marketing capabilities. The findings provide meaningful insights to researchers and bank managers on how to obtain sustainable competitive advances in the current digital era.

## 1. Introduction

The world is currently in the midst of the fourth industrial revolution (IR 4.0) that has been significantly transforming various traditional industries. With the rapid development of technologies, such as artificial intelligence (AI) and internet of things (IoT), an extremely large amount of big data (BD) is being produced anywhere and anytime, which provides great opportunities. BD has been considered “the next frontier for innovation, competition and productivity” (Manyika et al., 2011, p. 1) as it could be valuable assets to improve a company's competitiveness from operational, strategical, social, and even environmental aspects (Einav and Levin, 2014). Through big data analytics (BDA), an organization could facilitate business operations and marketing activities. Thus, BDA has become essential for an organization for business forecasting and strategic decision making (Ram and Zhang, 2022).

The Malaysian banking sector is considered stable while continue developing in facing rapidly changing market challenges along with regional and global financial crises in the last few decades (e.g., the 1997 Asian financial crisis and the 2008 global financial crisis) that may reoccur in the future. Meantime, as a country dependent on international trade the overall Malaysian economy and business activities is not immune to the health of global market and have been seriously disrupted by these crises. Amid the fast-changing and turbulent market environments, banks have to continuously modify their operations and strategies for sustainable competitive advantages. In other words, banks must conduct radical changes concerning organizational routines, processes, and practices to solve market challenges and explore potential opportunities. Banks are often considered as mature organizations, but they also face problems related to BD application (Cegarra-Navarro et al., 2019). Based on the extant literature, empirical research on BDA is still at an “infancy” stage, and it remains ambiguous what core resources and skills are needed for obtaining big data analytics capabilities (BDAC) (Mikalef et al., 2019). Therefore, there is an urgency to discuss what essential factors in constructing big data analytics capabilities (BDAC) to address a variety of new and emerging issues in the banking sector.

To survive in the unpredictable market, banks spare no efforts to provide customers with new products/services (e.g., mobile payment and facial recognition) and take on related organizational changes that improve value creation and customer experience optimization (Cegarra-Navarro et al., 2019). Organizations are able to gain competitive advantages by effectively analyzing big data from multiple internal and external channels. However, it is unclear how BDAC, as a dynamic ability, facilitates radical and incremental organizational changes (i.e., ambidexterity). The term ambidexterity is defined as an organization's ability to manage both exploitation and exploration-oriented activities simultaneously for long-term competitive advantages (Duncan, 1976). Significantly, there are contradictory tensions between exploitation and exploration as an organization only has limited resources within a certain period of time. The extant management literature reveals that there is a lack of discussion on how digital evolution improves organizational ambidexterity and solves the tensions between exploitation and exploration, especially in the banking sector.

**Figure 1:**
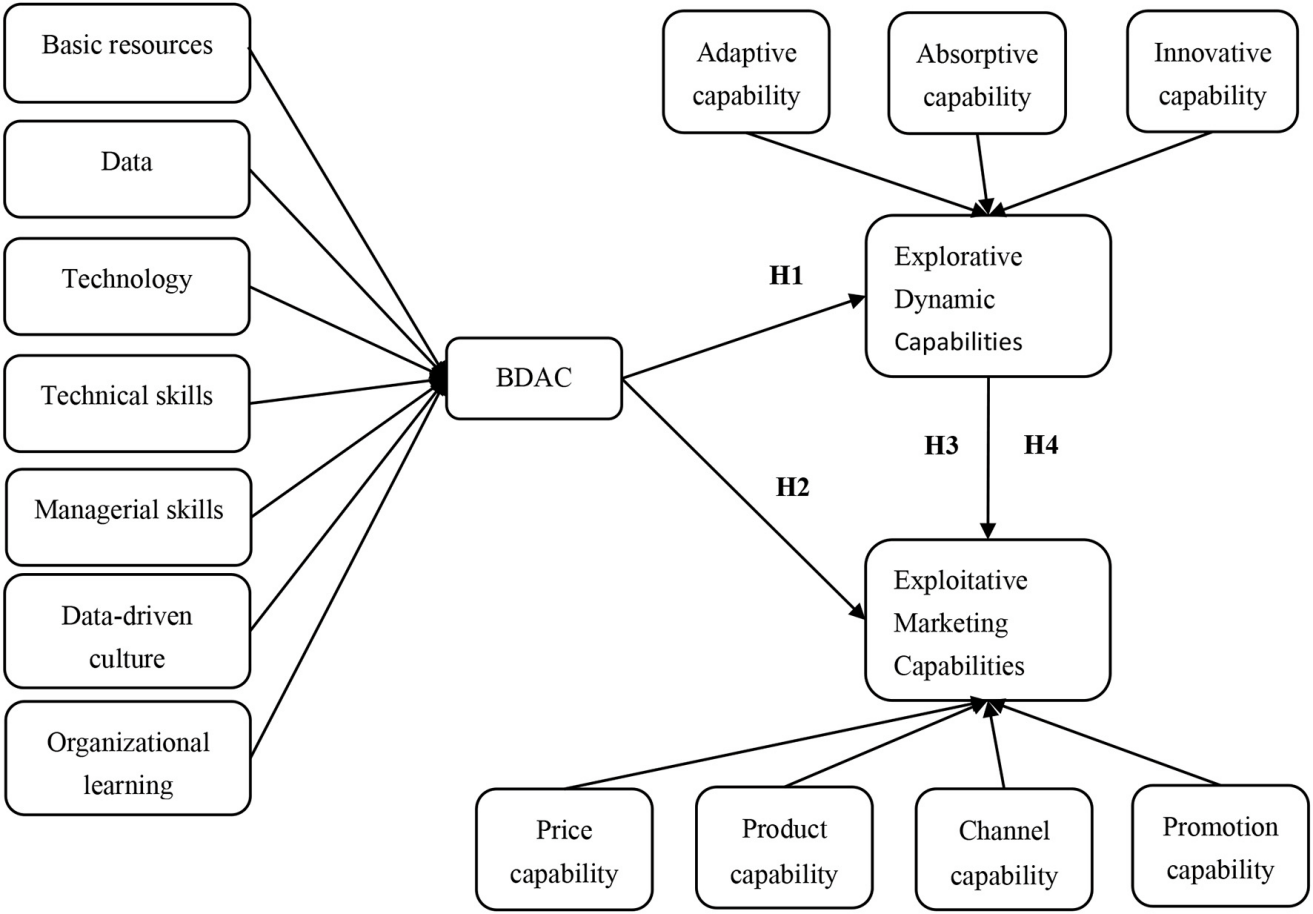
Conceptual framework and proposed hypotheses.

In an attempt to address these questions, this study is theoretically grounded on the dynamic capability view (DCV), which is elaborated in the next section. In the current intensified global competition, it is necessary to have a better understanding of dynamic capabilities for competitiveness (Ren and Peng, 2021). By addressing the research gaps, this research makes significant theoretical and practical contributions in the context of the Malaysian banking sector. The findings help banks obtain BDAC more effectively and efficiently, and understand the impacts of BDAC on organizational changes, which could facilitate banks achieving sustainable competitiveness in the financial market. This article is organized as follows: the next section describes the theoretical foundation for hypothesis and framework development (see Figure 1). Then, the proposed hypotheses are empirically examined by analyzing the collected data from bank managers. At the end, the article covers conclusions, limitations, and future research directions.

## 2. Literature review and hypothesis development

### 2.1. Dynamic capability view as the theoretical foundation

DCV is adopted as the theoretical foundation of the research for its effectiveness in explaining a firm's sustainable competitive advantages over competitors (Shams and Solima, 2019). DCV is an extension of the resource-based view (RBV) in strategic management (Teece et al., 1997). Barney (1991) proposed RBV as an important tool to understand how an organization solves obstacles and achieves competitiveness based on its configuration of internal resources. RBV is a valuable theory to illustrate the significance of managing in-house resources for a firm's competitive advantages (Erevelles et al., 2016). Nevertheless, RBV is considered as a static theory because it mainly focuses on a firm's internal resources, and neglects market's dynamic changes (Kraaijenbrink et al., 2010). On the contrary, DVC emphasizes an organization's ongoing evolutionary course on improving capabilities of adapting fast-changing situations by continuously enhancing its integration of internal and external resources amid an increasingly complex market environment (Teece et al., 1997; Gupta et al., 2019).

Besides, we could see that there are a couple of interrelated management philosophies being involved with DCV (Gupta et al., 2019; Shams and Solima, 2019). Firstly, the market conditions, ranging from macro to micro business environment, is a driving force to make a firm conceive its strategic goals. To achieve these goals, a firm must adjust its organizational structures and managerial processes. Secondly, a firm needs to allocate resources in line with its strategic goals aiming for competitive advantages within the market, which enables operational capabilities to roughly ascertain a firm's competitive position. Thus, dynamic capability is a concept of strategic management, which refers to an organization's abilities to strategically allocate, integrate and reconfigure its internal and external resources, in order to align its strategic goals with a dynamic market environment (Teece et al., 1997).

However, it should be noted that not all organizations are able to achieve their desired goals even if they could effectively deploy possessed resources synergistically toward their directed goals (Ghasemaghaei et al., 2017). To sustain competitive advantages, they must be agile and flexible enough to reconfigure their resources and skills in rapidly evolving conditions (Teece et al., 1997). DCV, as one of the most significant views in strategic management, provides organizations with perspectives to facilitate comprehending how dynamic capabilities could improve organizational resilience in adverse circumstances (Aljumah et al., 2021). Therefore, DCV is adopted as the grounded theory of the research, which justifies the necessity and appropriateness in linking BDAC and ambidexterity in the banking sector.

### 2.2. Big data analytics capabilities

The concept of big data was first introduced by Cox and Ellsworth in 1997, which is very different from the one of traditional data sets (Deshpande et al., 2019). The differences could be summarized in “5Vs,” namely volume, variety, velocity, veracity, and value (Wamba et al., 2017). The first feature refers to the extremely large quantity of data being generated; the second one refers to data heterogeneity with regard to different formats and sources; the third one refers to the exponentially growing rate of data generation; the fourth one refers to data quality being associated with the characters of the first “3Vs”; the last one refers to the worth of information that may provide economic, social and environmental benefits to stakeholders (Ferraris et al., 2019).

Not only does the “5Vs” model demonstrate the features of big data, it implies the importance of big data from different perspectives. In a review research conducted by Rabhi et al. (2019), its importance has been articulated in various business fields, such as business process management and human resources management. It is believed that big data will become even more crucial in the foreseeable future due to the wide application of digital technologies, such as the Internet of Things (IoT) (Ferraris et al., 2019). Significantly, it could be used as a potential resource for gaining sustainable competitive advantages (Wamba et al., 2017). Nevertheless, big data does not carry any business value without proper analysis. Thus, big data analytics (BDA), as a capability to collect, select, process, analyse, manage, and interpret big data for gaining values, is essential for an organization to extract business insights and even foresights from a voluminous data and translate these business values into sustainable market competitiveness (Wamba et al., 2017; Pappas et al., 2018; Ferraris et al., 2019).

To obtain big data analytics capabilities (BDAC), different resources and skills are required (Teece et al., 1997). The quality and quantity of big data is a primary foundation of BDAC, but managerial and technical issues matter as well in the process of BDAC formation (Gupta and George, 2016). Therefore, BDAC is broadly defined as a form of competence to gain meaningful business insights for sustainable competitive advantages by applying big data management, related technology infrastructure and talents (Kiron et al., 2014; Akter et al., 2016). Furthermore, Kiron et al. (2014) highlight the importance of establishing an analytics environment in which a firm's strategy matches its capability to outperform its competitors. Thus, BDAC is a layered concept being composed of various dimensions.

According to Akter et al. (2016), BDAC is composed of three main blocks, including management capability, technology capability and talent capability, which is supported by Davenport et al. (2012) as well. Specifically, Davenport and his colleagues state that the conceptualization of BDAC should focus on (a) big data management capability; (b) big data talents capability; and (c) tangible infrastructure capability. Besides, Ransbotham et al. (2015) argue that BDAC contains building blocks of management culture, data infrastructure, and data analytic skills. Furthermore, Wamba and Akter (2019) conclude that BDAC is a hierarchical construct, and each primary dimension is comprised by a few subdimensions. That is, BDAC is a construct with three dimensions (i.e., management capability, technology capability, and talent capacity), and each dimension has a few different subdimensions. Similarly, Mikalef et al. (2019) argue that BDAC consists of tangible resources, human skills, and intangible resources; tangible resources include data, technology and other basic resources; human skills contain technical and managerial skills; and intangible resources include data-driven culture and organizational learning.

### 2.3. Organizational ambidexterity

There are different definitions of organizational ambidexterity in the existing literature. Duncan (1976) is the first author to coin and introduce the concept of ambidexterity and ambidextrous organization in the domain of organizational management. Specifically, an ambidextrous organization has exploitative capabilities to address its present activities with high efficiency and has explorative capabilities to simultaneously address new challenges arising in the business environment (March, 1991). Later, Tushman and O'Reilly (1996) conceptualize ambidexterity as an organizational ability to implement both incremental and radical innovations responding to challenges at the same time. In one of the most recent studies, Monferrer Tirado et al. (2019) explain that ambidexterity is an organization's ability to balance conflicting objectives for aligning to the fast-changing environmental demands. According to the framework of organizational ambidexterity initially proposed by March (1991), there is a strong competition between exploitation and exploration activities within a firm.

Meanwhile, Eltantawy (2016) argues that there are contradictory tensions in developing exploitative and explorative capabilities, but an organization is less likely to achieve sustainable success if it fails to manage both exploitative and explorative activities simultaneously, which has been widely acknowledged in ambidexterity research (Peng and Lin, 2021a,b). Smith and Lewis (2011, p. 388) directly state that “Recent ambidexterity research has adopted a paradox lens, stressing that overall organizational success depends on exploring and exploiting simultaneously.” Besides, Zhou et al. (2021) state that exploration is the basis for organizational growth, but overemphasis on exploration will hinder exploitive capabilities that are indispensable for business operation. Therefore, the authors define ambidexterity as a dynamic capability of balancing both exploitative and explorative activities that allows an organization to sense market risks, identify new opportunities, and reconfigure resources accordingly.

Based on the relevant literature in relation to organizational management, exploitative and explorative capabilities are conceptually different (Cegarra-Navarro et al., 2019). March (1991) argues that exploration refers to activities linking with promoting innovation and discovering new possibilities from a future-oriented perspective, and exploitation, on the contrary, exploitation is associated with routine activities from a present-oriented perspective. Given the fact that a firm only has limited resources, there are many previous studies focusing on the dichotomy between a firm's exploitative and explorative goals concerning organizational ambidexterity (Montealegre et al., 2019). Due to the tensions between exploration and exploitation, a firm may find it difficult to balance between exploration ensuring future viability and exploitation ensuring current viability in the banking sector (March, 1991; Eltantawy, 2016; Monferrer Tirado et al., 2019). Nevertheless, Peng and Lin (2019) concluded that the tensions between exploration and exploitation cannot be entirely removed, and the most successful companies know how to reconcile the two kinds of capabilities for long-term competitiveness.

### 2.4. Big data analytics capabilities and ambidextrous capabilities

Our society is now becoming closely connected because of the development of modern information and communication technologies, which generates a large quantity of heterogeneous and complex data sets (i.e., big data) (Buhalis and Law, 2008; Ferraris et al., 2019). As a result, a firm is required to attain a more efficient and sustainable managerial approach for making strategic decisions to gain competitive advantages in a complex market context (Caputo et al., 2021), which explains the necessity to develop BDAC for banks.

It has been mentioned that BDAC are composed of tangible resources, human skills, and intangible resources (Mikalef et al., 2019). Significantly, BDAC-related human skills and intangible resources are associated with a firm's organizational routines (Aljumah et al., 2021). This research is mainly established on the dynamic capability view (DCV) highlighting an organization's abilities to adapt to the ever-changing environment through continuous improvements of resources integration, process optimization and strategy adjustment (Teece et al., 1997). BDAC-related dynamic capabilities are directly linked with organizational routines and practices (Teece, 2018). By modifying routines, practices and resources for data efficiency, an organization (e.g., bank) could solve current difficulties and identify new opportunities (Mikalef et al., 2019; Aljumah et al., 2021). Specifically, improvement of information management could facilitate organizations addressing current threats and new challenges (Monferrer Tirado et al., 2019). Therefore, the following hypotheses are proposed:

H1: BDAC positively influences a firm's explorative capabilities in the banking sector of Malaysia.
H2: BDAC positively influences a firm's exploitative capabilities in the banking sector of Malaysia.

As mentioned earlier, there are contradictory tensions between exploration and exploitation within an organization. To solve the tensions, a firm has to obtain abilities to conduct different even conflicting activities at the same time (Monferrer Tirado et al., 2019). Thus, “Recent ambidexterity research has adopted a paradox lens, stressing that overall organizational success depends on exploring and exploiting simultaneously” (Smith and Lewis, 2011, p. 388). That is, both exploitative and explorative capabilities are necessary for organizational development. Zahra et al. (2006) argue that explorative capabilities are one of the most important factors influencing competitive advantages. When a firm becomes more adaptive, absorptive and innovative by radically improving its organizational routines, processes and practices, it understands customers, competitors, and other stakeholders more. Then, it could transform these deep market-oriented understandings into suitable products and services. Significantly, Monferrer Tirado et al. (2019) find that exploitative capabilities have a positive effect on explorative capabilities in the banking sector. Thus, the following hypotheses are proposed:

H3: A firm's explorative capabilities positively influences its exploitative marketing capabilities in the banking sector of Malaysia.
H4: A firm's explorative capabilities mediates the positive relationship between BDAC and exploitative marketing capabilities in the banking sector of Malaysia.

## 3. Methodology

All the measurement items of the research are adopted from previous studies and tailored to the banking sector. The questionnaire items are measured on a 7-point Likert scale, where “1” represents “completely disagree” and “7” represents completely agree. The survey is arranged into three sections: the first one includes 25 items measuring BDAC with seven dimensions from Mikalef et al. (2019); the second one contains a few descriptive questions for banks (e.g., business type and category); the third one includes 11 items measuring explorative capabilities with three dimensions; and 16 items measuring exploitative capabilities with four dimensions from Monferrer Tirado et al. (2019). The rationale to place descriptive questions between BDAC and explorative and exploitative capabilities is to reduce the impact of common method bias (Podsakoff et al., 2012).

The targeted respondents of the research are managers from banks based in Malaysia that have adopted big data analytics (BDA) because they arguably know their banks' arrangement for data-related technological development and they also participate in improvement of organizational routines, processes, and practices. To ensure that the respondents are desired, two filter questions are added in the survey. The first one asks, “Does your bank use BDA?” and the second one asks, “Are you a bank manager?”. Before data collection, two rounds of pre-test are conducted with five bank managers to make sure that the respondents understand the survey the same way as the researchers do. Based on their feedback, a few items are modified. Then, the modified survey is finalized by three academic scholars in organizational management. To target the right potential respondents for the research, a snowball sampling method is used (Aziz and Long, 2022). Initially, the questionnaire was distributed to 10 senior bank managers who work at various banks based in Malaysia via the leading author's personal network. Then, the 10 senior bank managers sent out the surveys to their colleagues. From 1 October 2021 to 1 December 2021, 200 questionnaires were sent to bank managers. By the end of December 2021, we have received 162 valid responses. Both G^*^Power software and “ten times rule” are used, and it is confirmed that the number of responses exceeds the minimum sample size requirement for data analysis (Chin, 2010; Hair et al., 2021).

## 4. Data analysis

The partial least squares structural equation modeling (PLS-SEM) technique was adopted for data analysis (Ringle et al., 2015). PLS-SEM is an alternative approach to covariance-based structural equation modeling (CB-SEM) technique. The former one aims to maximize the explained variance of endogenous latent variables, and the latter one aims to reproduce theoretical co-variance matrix without focusing on explaining variance. In social sciences, especially business research, PLS-SEM is widely applied for various advantages (Hair et al., 2021). Concerning this research, PLS-SEM is chosen rather than CB-SEM mainly because CB-SEM is typically used for reflective constructs only and the current research has three 2nd order formative constructs (Hair et al., 2021).

Regarding size requirement for data analysis and hypothesis testing, this research used G^*^Power to calculate the minimum sample size (Hair et al., 2021). By conducting linear multiple regression, it is found that the minimum sample size of the research is 64 with a power at 0.95. Therefore, 162 valid responses surpass the minimum requirement of sample size.

### 4.1. Measurement model assessment

To test the proposed hypotheses, the current research has to confirm the measurement and structural measurement models (Hair et al., 2021). The three latent variables of the research framework are reflective-formative 2nd order constructs. That is, these three constructs are measured by various dimensions (formative 2nd order), and each dimension is measured by different reflective indicators (reflective 1st order). Thus, divergent assessment criteria were applied to evaluate reflective dimensions and formative constructs.

To assess the reflective measurement model (seven 1st order dimensions of BDAC, three 1st order dimensions of EDC and four 1st order dimensions of EMC), internal reliability, convergent validity and discriminant validity have to be confirmed. As shown in Table 1, factor loadings, composite reliability (CR), and average variance extracted (AVE) values of all the 1st order reflective dimensions surpass the cut-off thresholds at 0.7, 0.7, and 0.5 respectively. Therefore, the reliability and convergent validity of all the 1st order reflective dimensions are confirmed.

**Table 1:** Validation of the measurement scales.

**Construct/dimension**	**Type**	**Item**	**Loading**	**CR**	**AVE**
Basic resources	Reflective	BR1	0.929	0.946	0.915
		BR2	0.914		
Data	Reflective	DA1	0.918	0.932	0.82
		DA2	0.893		
		DA3	0.904		
Technology	Reflective	TE1	0.897	0.943	0.805
		TE2	0.893		
		TE3	0.891		
		TE4	0.908		
Technical skills	Reflective	TS1	0.913	0.937	0.914
		TS2	0.915		
		TS3	0.915		
		TS4	0.901		
Managerial skills	Reflective	MS1	0.915	0.948	0.916
		MS2	0.927		
		MS3	0.908		
		MS4	0.918		
Data-driven culture	Reflective	DC1	0.751	0.884	0.655
		DC2	0.832		
		DC3	0.812		
		DC4	0.840		
Organizational learning	Reflective	OL1	0.925	0.941	0.831
		OL2	0.922		
		OL3	0.904		
		OL4	0.873		
Adoptive capability	Reflective	AAC1	0.923	0.936	0.878
		AAC2	0.942		
		AAC3	0.916		
Absorptive capability	Reflective	AOC1	0.943	0.944	0.874
		AOC2	0.923		
		AOC3	0.930		
Innovative capability	Reflective	IC1	0.895	0.945	0.796
		IC2	0.859		
		IC3	0.916		
		IC4	0.886		
		IC5	0.904		
Pricing capability	Reflective	PC1	0.947	0.941	0.866
		PC2	0.908		
		PC3	0.927		
Commercialization capability	Reflective	CC1	0.792	0.909	0.769
		CC2	0.926		
		CC3	0.908		
Channel management capability	Reflective	CMC1	0.848	0.937	0.788
		CMC2	0.916		
		CMC3	0.872		
		CMC4	0.912		
Communication capability	Reflective	COC1	0.912	0.946	0.785
		COC2	0.904		
		COC3	0.900		
		COC4	0.920		
		COC5	0.855		
		COC6	0.819		

Then, the discriminant validity of the 1st order reflective dimensions of BDAC, EDC and EMC are examined by assessing their Heterotrait-Monotrait of correlations (HTMT) values (Henseler et al., 2015). As shown in Table 2, none of the HTMT scores exceed the cut-off threshold at 0.9, so the discriminant validity of the 1st order reflective dimensions is confirmed (Gold et al., 2001).

**Table 2:** Discriminant validity (HTMT).

**Construct**	**AAC**	**AOC**	**BR**	**CC**	**CMC**	**COC**	**DA**	**DC**	**IC**	**MS**	**OL**	**PC**	**TE**
AOC	0.835												
BR	0.704	0.763											
CC	0.648	0.799	0.602										
CMC	0.722	0.820	0.631	0.883									
COC	0.753	0.824	0.588	0.892	0.896								
DA	0.831	0.849	0.885	0.688	0.721	0.753							
DC	0.744	0.800	0.605	0.731	0.812	0.72	0.684						
IC	0.876	0.895	0.733	0.711	0.862	0.825	0.818	0.796					
MS	0.832	0.825	0.832	0.718	0.786	0.782	0.881	0.679	0.843				
OL	0.174	0.421	0.220	0.447	0.423	0.353	0.26	0.452	0.357	0.258			
PC	0.754	0.687	0.697	0.732	0.727	0.698	0.778	0.537	0.77	0.726	0.135		
TE	0.569	0.762	0.727	0.718	0.602	0.557	0.804	0.585	0.591	0.696	0.379	0.499	
TS	0.822	0.798	0.818	0.648	0.663	0.663	0.85	0.653	0.679	0.852	0.273	0.652	0.695

Discriminant validity established at HTMT_0.90_.

**Figure 2:**
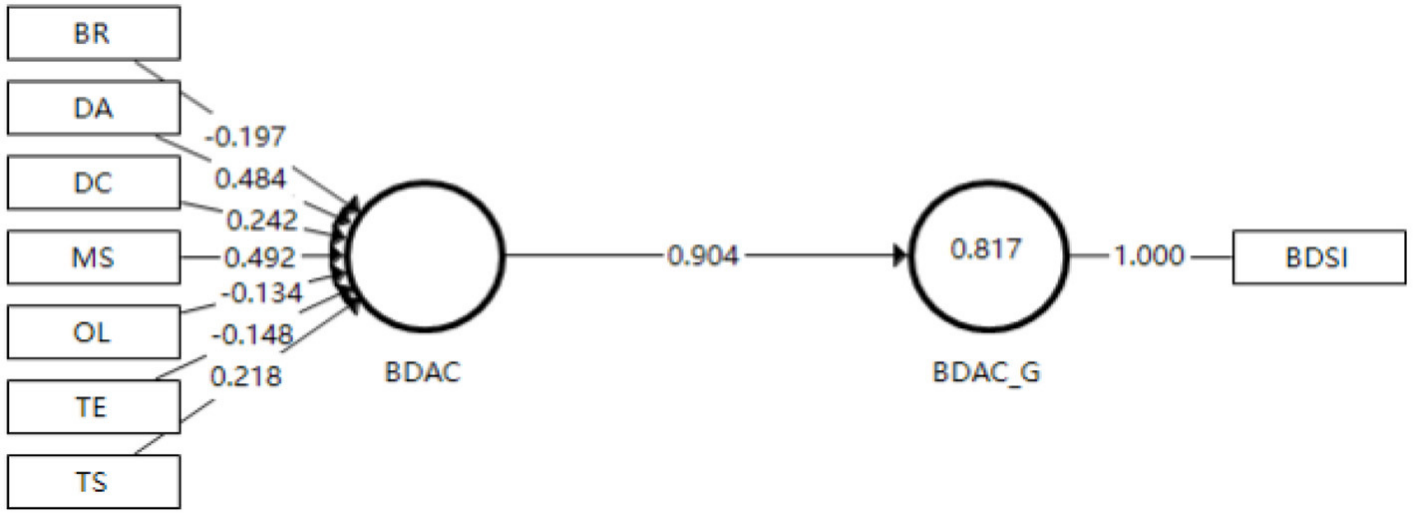
Results of redundancy analysis (BDAC).

**Figure 3:**
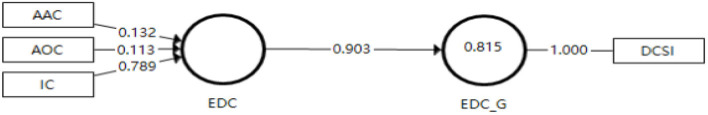
Results of redundancy analysis (EDC).

**Figure 4:**
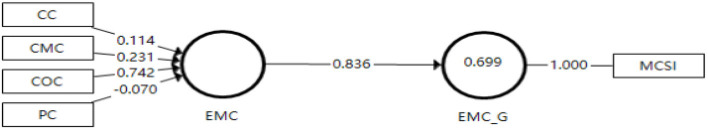
Results of redundancy analysis (EMC).

BDAC, EDC and EMC are reflective-formative 2nd order composite constructs. To assess the formative measurement model, convergent validity, collinearity, and significance of formative indicators/dimensions have to be examined (Hair et al., 2021). With regard to the three formative constructs' convergent validity, they are assessed via redundancy analysis (Chin, 1998). According to Figures 2–4, the path coefficient between the formative constructs (i.e. BDAC, EDC and EMC) and the same constructs that are reflectively measured by a global single item are all much higher than the threshold value at 0.70. Thus, the convergent validity of the three formative constructs is confirmed (Hair et al., 2021).

Concerning potential collinearity issues, it is examined by looking at variance inflation factor (VIF) (Hair et al., 2021). For formative constructs, indicators/dimensions are not inter-changeable, so high correlations are not desired. Specifically, high correlations among a formative construct's indicators/dimensions can distort estimation of outer weights (Hair Jr et al., 2014). According to Table 3 below, none of the VIF scores of formative constructs' dimensions are higher than 5. Therefore, BDAC, EDC and EMC are not very likely to encounter a collinearity problem (Hair et al., 2011).

**Table 3:** Convergent validity (2nd order formative construct).

**Composite (2nd order construct)**	**1st order dimension**	**Weight**	***T*-value**	***P*-value**	**CI**	**VIF**
Big data analytics capabilities	BR	−0.062	0.879	0.190	−0.178, 0.059	3.659
	DA	0.365	3.385	0.000	0.196, 0.546	4.865
	TE	−0.094	1.338	0.091	−0.226, 0.010	2.391
	TS	−0.008	0.064	0.475	−0.183, 0.204	3.851
	MS	0.555	5.806	0.000	0.390, 0.706	4.411
	DC	0.309	4.354	0.000	0.186, 0.414	1.856
	OL	0.062	1.201	0.115	−0.016, 0.152	1.265
Explorative dynamic capabilities	AAC	0.288	3.856	0.000	0.162, 0.406	3.205
	AOC	0.284	2.481	0.007	0.095, 0.475	4.206
	IC	0.488	3.935	0.000	0.283, 0.686	4.850
Exploitative marketing capabilities	PC	0.350	3.147	0.001	0.143, 0.500	1.964
	CC	−0.106	0.607	0.272	−0.373, 0.189	3.660
	CMC	0.404	3.200	0.001	0.195, 0.617	4.748
	COC	0.442	3.524	0.000	0.237, 0.653	4.595

CI, confidence intervals; VIF, variance inflation factor.

Then, the significance and relevance of formative indicators/dimensions have to be evaluated by conducting a bootstrapping procedure to estimate the outer weights of the three formative constructs' dimensions (Hair et al., 2021). Outer weight is considered an important criterion for assessing the relative contribution of a formative indicator/dimension, and *T*-value, *P*-value and confidence intervals are looked at to check whether outer weight is significant (Gannon et al., 2021). As shown in Table 3, the outer weights of four BDAC's dimensions and one EMC's dimension are not significant. Nevertheless, Table 4 indicates that the five dimensions' outer loadings are all higher than 0.5. Therefore, the five dimensions are maintained even if their outer weights are not significant (Hair et al., 2021).

**Table 4:** Outer loading (formative dimensions).

	**BDAC**	**EDC**	**EMC**
AAC		0.908	
AOC		0.930	
BR	0.778		
CC			0.815
CMC			0.936
COC			0.936
DA	0.891		
DC	0.801		
IC		0.970	
MS	0.941		
OL	0.562		
PC			0.840
TE	0.670		
TS	0.827		

### 4.2. Structural model assessment

To assess the structural model, *t*-value, *P*-value, confidence intervals, coefficient of determination (*R*^2^), effect size (*f*^2^) and predictive relevance (*Q*^2^) are evaluated (Hair et al., 2021). By conducting a bootstrapping technique with 5,000 resamples, whether the structural model relationships are significant are examined. According to Table 5, H1, H2, and H3 are supported at 95% confidence intervals. Firstly, BDAC positively influences explorative dynamic capabilities (β = 0.598, *t* = 5.375, *p* = 0.000, LL = 0.479, CL = 0.523); BDAC positively influences exploitative marketing capabilities (β = 0.391, *t* = 3.799, *p* = 0.000, LL = 0.254, CL = 0.586); explorative dynamic capabilities positively influence exploitative marketing capabilities (β = 0.511, *t* = 4.919, *p* = 0.000, LL = 0.304, CL = 0.648); and explorative dynamic capabilities mediate the positive relationship between BDAC and exploitative marketing capabilities (β = 0.459, *t* = 4.888, *p* = 0.000, LL = 0.273, CL = 0.585) in the banking sector of Malaysia.

**Table 5:** Results summary for the hypothesis testing.

**Hypothesis**	**Relationship**	**Beta**	**SD**	***T*-Value**	***P*-value**	**LL**	**UL**	**Decision**
H1	BDAC -> EDC	0.598	0.014	5.375	0.000	0.479	0.523	Supported
H2	BDAC -> EMC	0.391	0.103	3.799	0.000	0.254	0.586	Supported
H3	EDC -> EMC	0.511	0.104	4.919	0.000	0.304	0.648	Supported
H4	BDAC -> EDC -> EMC	0.459	0.094	4.888	0.000	0.273	0.585	Supported

LL, lower limit; UL, upper limit at 95 percent confidence intervals.

Besides, the model's predictive accuracy is evaluated by looking at the coefficient of determination (*R*^2^). As shown in Table 6, the *R*^2^ scores of explorative dynamic capabilities and exploitative marketing capabilities are 0.507 and 0.473, indicating BDAC has a substantial level of predictive accuracy on the two endogenous latent variables (Cohen, 2013). Then, effect size (*f*^2^) is checked to assess the relative impact of BDAC (predicting variable) on the two endogenous latent variables. It is shown that the *f*^2^ values of BDAC on explorative dynamic capabilities and exploitative marketing capabilities are 0.374 and 0.130, and the *f*^2^ values of explorative dynamic capabilities on exploitative marketing capabilities is 0.223, which indicates a satisfactory effect size (Cohen, 2013). Lastly, Stone and Geisser' *Q*^2^ is looked at via a blindfolding technique to assess the predictive validity of the path model. Table 6 shows that the *Q*^2^ scores are higher than 0, so BDAC has a predictive relevance on explorative dynamic capabilities and exploitative marketing capabilities (Hair et al., 2021). Concerning model fit, a test of standardized root mean square residual (SRMR) is conducted. It is found that the SRMR score (0.045) of the model is lower than the suggested threshold at 0.08. Thus, this research has a good fit of the PLS path model (Henseler et al., 2014).

**Table 6:** Assessment of *R*^2^, *f*^2^, and *Q*^2^.

	***R* ^2^**	***f*** ^**2**^		***Q* ^2^**
		**EDC**	**EMC**	
BDAC		0.374	0.130	
EDC	0.507		0.223	0.525
EMC	0.473			0.547

## 5. Conclusions and discussion

This research examines the effects of big data analytics capabilities (BDAC) on organizational ambidexterity and the relationship between exploration and exploitation in the context of the Malaysian banking sector. We attempt to fill these gaps by addressing the two research questions: (1) how banks configure core resources/skills to acquire BDAC and organizational ambidexterity; and (2) how digital evolution (i.e., BDAC) effects organizational changes (i.e., ambidexterity).

The results of this study suggest that BDAC, explorative dynamic capabilities (EDC) and exploitative marketing capabilities (EMC) are all multifaceted constructs with different dimension, BDAC positively influences organizational ambidexterity (i.e. EDC and EMC), and EDC also mediates the relationship between BDAC and EMC, which facilitates banks obtaining sustainable competitive advantages in the turbulent financial market by highlighting digital evolution and organizational changes.

We can conclude that banks should develop BDAC in the era of digital revolution for gaining market competitiveness, and banks could speed up changes in organizational routines, processes and practices that are key factors to handle the exiting challenges and exploring new opportunities if they actively and effectively pursue BDAC. Besides, they are likely to resolve the contradictory tensions between exploration and exploitation. Therefore, it is imperative to develop both BDAC and organizational ambidexterity from the lens of dynamic capability view (DCV).

### 5.1. Theoretical contributions

BDAC and organizational ambidexterity have been widely discussed in the business domain, but their application and interplay are less explored in the banking sector because banks are usually considered as mature organizations. Nevertheless, banks also have to proactively embrace digital evolution and organizational changes for sustainable competitiveness in the unpredictable financial market environments (Cegarra-Navarro et al., 2019). We make contributions to the information system management strategic management in the following ways.

First, we extend the relevant literature on dynamic capability view (DCV) from a perspective of big data application in the banking sector. According to DVC, an organization has to keep improving adaptation abilities through continuous configuration of its resources, skills, and expertise amid ever-changing environments (Teece et al., 1997). However, the pertinent literature does not sufficiently explore how to develop BDAC. To facilitate making better strategic decisions in the current digital era, banks and other organizations have to have a deep understanding of what key resources and skills are required for BDAC. The current research confirms that tangible resources, human skills, and intangible resources are essential to establish BDAC in the banking sector (Mikalef et al., 2019). By identifying the key factors, banks could allocate, integrate, and reconfigure their resources, skills and expertise more effectively and efficiently for competent BDAC so as to achieve competitive status in a dynamic and turbulent market environment (Teece et al., 1997).

Second, we advance the prior literature by confirming an association between BDAC and organizational ambidexterity (i.e., EDC and EMC). To develop BDAC, organizations (e.g., banks) have to allocate, integrate and reconfigure various resources and skills. Some resources and skills for digital evolution, such as data-driven culture and organizational learning, are closely related to dynamic adaptation abilities in unpredictable situations and conditions concerning organizational routines, processes, and practices (Aljumah et al., 2021). Significantly, these dynamic adaptation abilities (a.k.a. organizational ambidexterity) are very crucial for banks to maintain their sustainable competitive advantages in the turbulent financial market. Our findings suggest that BDAC positively influences explorative activities (EDC) and exploitative activities (i.e., EMC). Thus, banks are more likely to address the existing challenges and identify new opportunities by enhancing their BDAC.

Third, we clarify the interplay between exploration and exploitation within the framework of organizational ambidexterity. As per the ambidexterity theory, there are inherent tensions between explorative and exploitative activities due to the limited resources within an organization. Nevertheless, we have to see organizational ambidexterity from a paradoxical lens highlighting that a firm's overall success depends on both exploration and exploitation and how to balance the tensions between the two aspects of organizational ambidexterity. Our findings reveal that EDC positively influences EMC, and EDC mediates the positive relationship between BDAC and EMC, which reaffirms the central role of EDC for a firm's long-term success. Therefore, this research makes a significant contribution to the existing literature pertinent to organizational ambidexterity by further clarifying the paradoxical relationship between exploration and exploitation in the banking sector, proving that exploration and exploitation should be pursued simultaneously, and revealing that exploration facilitates exploitation.

### 5.2. Practical implications

Based on the theoretical contributions, some recommendations are provided for managers of the service industry, particularly in the banking sector. Banks should integrate big data from both internal and external sources for high-value analysis as competent data analysis may provide banks with unique advantages over competitors by providing valuable operational and marketing insights. Given the importance of BDAC, bank managers are advised to pay extra attention to big data, managerial skills, and data-driven culture as the three resources/skills are very vital for developing BDAC. With sufficient BDAC, banks are able to analyse various current phenomena, such as customer purchase behavior and competitors' decisions. Then, they could predict market trends, new opportunities, and competitors' next moves (Pappas et al., 2018). In Customer Relationship Management (CRM), BDAC also facilitates customer value generation and maximization. By setting customer value as an underlying goal, banks are likely to increase their profitability through targeting the right customers, improve customer experience, maintaining good customer relationships and reducing the cost of customer acquisition/retention. Thus, banks are advised to treat BDAC seriously, and managers should make strategic decisions based on the combination of data-driven insights and personal experience.

From the perspective of organizational management, banks also need to proactively embrace BDAC as they are operating in a dynamic, complex, and turbulent financial market. To address current threats and future opportunities, banks should pursue both exploitation and exploration simultaneously. More significantly, exploration-oriented activities also facilitate exploitation-oriented activities. Meanwhile, exploration mediates the relationship between BDAC and exploitation. Thus, a bank is advised to keep a balance between explorative dynamic capabilities (EDC) and exploitative activities (i.e., EMC) in line with the market conditions. Overemphasis either EDC or EMC will hinder banks achieving sustainable competitive advantages. Besides, a bank should allocate extra efforts (e.g., resources and skills) toward explorative dynamic capabilities (EDC) if it is highly interested in developing new ideas and thoughts for introducing new products/services as these innovations require radical changes concerning organizational routines, processes, and practices.

## 6. Limitations and future research directions

This research makes significant theoretical and practical contributions, but it is not free of limitations. First, the study is conducted in a specific service industry (i.e., Malaysian banks sector). There are considerable differences even within the same services industry, so future research are advised to make comparisons across different service sectors, which may further enhance our understanding of BDAC and ambidexterity in the whole services industry. Second, there might be other dimensions of BDAC, EDC and EMC that exceed the identified scope of the research. Thus, future studies should take other possible dimensions into consideration to better conceptualize the three composite constructs. Third, all the measurement items are adopted/adapted from prior studies to the banking sector. There may be extensions of existing measures. Future research could develop specific measures to banks in the context of BDAC and ambidexterity. Fourth, this research collects cross-sectional data from bank managers, which may only reflect the respondents' short-term perception on BDAC and organizational ambidexterity. Therefore, future studies are advised to collect longitudinal data for comparison. Similarly, the respondents include branch managers, mid-level, and top-level managers, but data numbers of the three categories are not sufficient for statistical comparison. Thus, future studies could collect more data from the three management levels and compare whether there are similarities and differences in the relationship between BDAC and organizational ambidexterity, and the association between exploration and exploitation.

## Data availability statement

The raw data supporting the conclusions of this article will be made available by the authors, without undue reservation.

## Ethics statement

Ethical review and approval was not required for the study on human participants in accordance with the local legislation and institutional requirements. The patients/participants provided their written informed consent to participate in this study.

## Author contributions

NA: supervision, research idea, data collection, and fund. FL: research idea, conceptualization, and writing—original draft preparation. All authors contributed to the article and approved the submitted version.
